# Extensive Bilateral Pleural Plaques: A Cadaveric Case Study

**DOI:** 10.7759/cureus.107079

**Published:** 2026-04-15

**Authors:** Mathangi Rajaram-Gilkes, Renee Frank

**Affiliations:** 1 Medical Education, Geisinger Commonwealth School of Medicine, Scranton, USA; 2 Anatomic and Clinical Pathology, Geisinger Community Medical Center, Scranton, USA

**Keywords:** asbestos induced pneumonia, asbestosis, basketweave collagen bundles, bilateral diaphragmatic pleural thickening, diaphragmatic pleural plaques, histology of pleural plaques, mesothelioma, pleural adhesions, pleural calcification, pleural plaques

## Abstract

Pleural plaques are the most common manifestation of asbestos-related pleural disease and function primarily as markers of prior exposure rather than as direct causes of clinically significant symptoms, often appearing several decades after initial contact. They are typically asymptomatic and discovered incidentally on imaging studies or at autopsy, and they initially present as small, well-circumscribed lesions along the parietal pleura, most commonly over the rib surfaces while sparing the intercostal spaces. They may progressively enlarge, coalesce, and undergo calcification over time. Although asbestos exposure is the primary etiology, pleural plaques and pleural calcifications may also arise from prior infections such as tuberculosis or empyema, trauma, hemothorax, end-stage renal disease, or chronic inflammatory and autoimmune conditions such as systemic lupus erythematosus and rheumatoid arthritis, and in some instances they may be idiopathic. Additionally, pleural plaques can be associated with mesothelioma. When extensive, pleural plaques may restrict lung expansion, leading to exertional dyspnea that can progress to dyspnea at rest and, in advanced cases, respiratory insufficiency.

During routine cadaveric dissections conducted as part of the preclinical medical curriculum at the Geisinger Commonwealth School of Medicine (GCSOM), Scranton, Pennsylvania, extensive bilateral pleural thickening, predominantly involving the diaphragmatic pleura, was observed in an 89-year-old male cadaver. After the opening of the thoracic cage and removal of the lungs and heart, pleural plaques were identified, examined, and measured. The pleural cavities were relatively free, with minimal adhesions involving the bases of both lungs in the diaphragmatic regions, and any evidence of pleural effusion was likely lost during the dissection process. Small, isolated pleural plaques were also noted along the anterior aspect of the right parietal costal pleura. A small, firm mass was palpated within the anterior border of the right upper lobe. Tissue specimens were obtained from the pleural plaques of the anterior chest wall and diaphragmatic pleura, as well as from the pulmonary mass, for histopathological evaluation. Microscopic examination demonstrated abundant fibrocollagenous thickening of the parietal pleura with adjacent fibro-adipose connective tissue and associated inflammation, without evidence of asbestos fibers. Histological analysis of the pulmonary mass was consistent with acute pneumonia.

Pleural plaques are frequently encountered as incidental findings on chest radiographs and computed tomography scans in elderly individuals, particularly those with prior asbestos exposure, yet many such findings remain clinically uninvestigated during life. Establishing an etiology in cadaveric cases poses a significant challenge due to the absence of reliable occupational, environmental, and medical histories. While the histopathologic features observed in this case are consistent with previously published descriptions, the gross anatomical documentation of extensive pleural plaques predominantly confined to the diaphragmatic pleura, including detailed measurements, texture, and imaging, has not been previously reported. Dissemination of these findings aims to enrich anatomical and pathological educational resources, support future research, and deepen the understanding of pleural pathology encountered during cadaveric dissection and medical education.

## Introduction

The pleura is a thin, transparent, two-layered membrane that covers the lungs (visceral layer) and lines the inside of the chest wall (parietal layer). The layer that covers the lung is continuous with the parietal layer at the hilum of the lung, forming the root of the lung. Between the two thin, flexible layers is a small amount of fluid (pleural fluid) that lubricates them as they slide smoothly over one another with each breath. The area containing the fluid is referred to as the pleural space [1]. The pleura, which is normally very thin and flexible, sometimes becomes thick as a result of a reactive change (fibrosis) due to inflammation or as a result of asbestos exposure (called asbestos-related pleural disease). Most of the time, only a small area of the pleura is affected, while at other times, larger areas are involved. The fibrotic pleura can also develop calcification (accumulation of calcium within the tissue). The causes of these reactive changes appearing as plaques are most commonly described in the literature as secondary to asbestosis [2]. Pleural thickening observed during the dissection of a cadaver in our lab, along with the corresponding histopathological findings, is presented and discussed in this report.

## Case presentation

During the preclinical phase of medical education at Geisinger Commonwealth School of Medicine (GCSOM) in Scranton, cadaveric dissection was performed on an 89-year-old male donor who had worked for many years as a steam fitter in Union, New Jersey, USA. The dissection took place during the cardiovascular and pulmonary block, which followed completion of the gastrointestinal system block. Accordingly, the abdominal cavity was dissected first, followed by the thoracic region. Examination of the exterior thoracic cage revealed steel wire sutures securing the sternum at the midline, consistent with a prior thoracotomy. The thoracic cage was opened along the midaxillary lines from the level of the diaphragm and extended to the clavicles, and the anterior chest wall was reflected anteriorly after detachment at the sternoclavicular joints, exposing the mediastinum and pulmonary cavities. The heart was observed to be enlarged and demonstrated two coronary artery bypass grafts. Notably, the left internal thoracic artery was absent from its expected location within the left transversus thoracis muscle, suggesting its use as a conduit for grafting. The inferior surface of the heart rested on the diaphragm within the normal tendinous portion. Both lungs were subsequently removed along with their visceral pleural reflections for further examination.

The parietal and visceral layers of the pleura were carefully examined. The right pleural cavity contained a small amount of fluid compared to the left. Gross examination of the right lower lobe revealed erythematous changes with a mild collapse of the lung tissue, suggestive of an underlying infectious process. A small, firm, palpable nodular lesion was identified within the anterior border of the right upper lobe. Bilaterally, the diaphragmatic pleura demonstrated opaque, whitish plaques at sites corresponding to the areas where the lung bases rested on the diaphragm. These plaques appeared glassy in nature and were moderately pliable and reasonably flexible to palpation, involving the muscular portions of the diaphragm. Additional small patches of whitish plaques were observed on the inner aspect of the anterior chest wall beneath the right parietal costal pleura around the third and fourth intercostal spaces. The diaphragmatic plaques and the largest anterior chest wall lesions were measured, and respective sections were obtained for histopathological analysis. A specimen was also collected from the firm pulmonary mass at the interface between the lesion and adjacent grossly normal lung tissue. Histopathologic evaluation was performed at Geisinger Community Medical Center in collaboration with the pathology team, and the resulting images are hereby presented.

Cadaveric findings

Thoracic Cage

**Figure 1:**
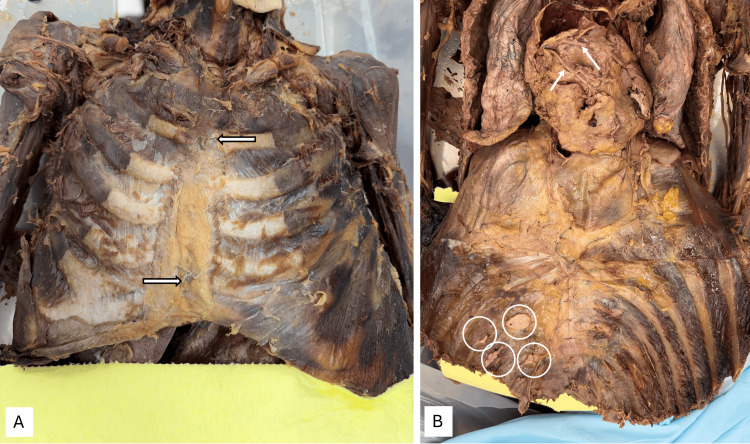
External findings Image A shows steel wires indicating thoracotomy. Image B shows a reflected anterior thoracic wall with pleural plaques (white circles). An enlarged heart with two coronary bypass grafts can be seen (white arrows)

External findings showed intermittent steel wires running through the manubrium and body of the sternum, indicating a prior thoracotomy (Figure 1A). Figure 1B shows the reflected anterior thoracic wall, revealing an enlarged heart and two coronary artery bypass grafts (white arrows). The absence of the left internal thoracic artery on the anterior chest wall was also noted. The collapsed appearance of the lower lobe of the right lung was observed. On the anterior chest wall, there were four small, isolated plaques on the parietal pleura, with their locations indicated by white circles.

**Figure 2:**
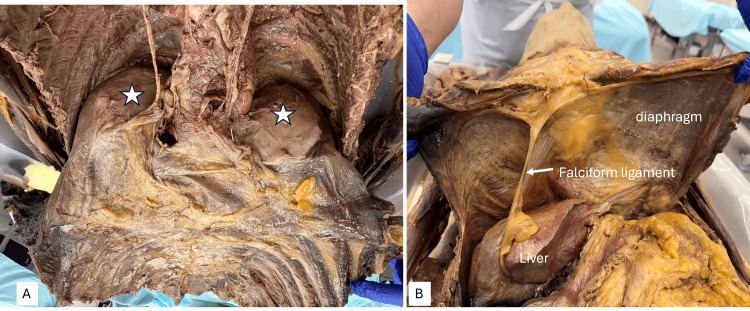
Overview of the diaphragm - image 1 Image A shows the plaques (indicated by the stars). Image B shows a view of the diaphragm from the abdomen, showing the liver, falciform ligament, and the muscular portions of the diaphragm. The abdominal aspect shows no plaques

Figure 2A shows an image in which both lungs had been removed. Bilateral whitish, glassy plaques on the parietal diaphragmatic pleura in areas (marked by the stars) related to the posterior aspects of the bases of both lungs were observed. They were smooth to palpate, with a firm, partially calcified texture, and were moderately pliable. Figure 2B shows a view of the underside of the diaphragm from the abdomen. The thoracic cage was detached along the costal margin and elevated to view the diaphragm from the abdomen. The liver, falciform ligament, and the muscular portions of the diaphragm were clearly visible, and there was no evidence of whitish plaque infiltration on this aspect.

**Figure 3:**
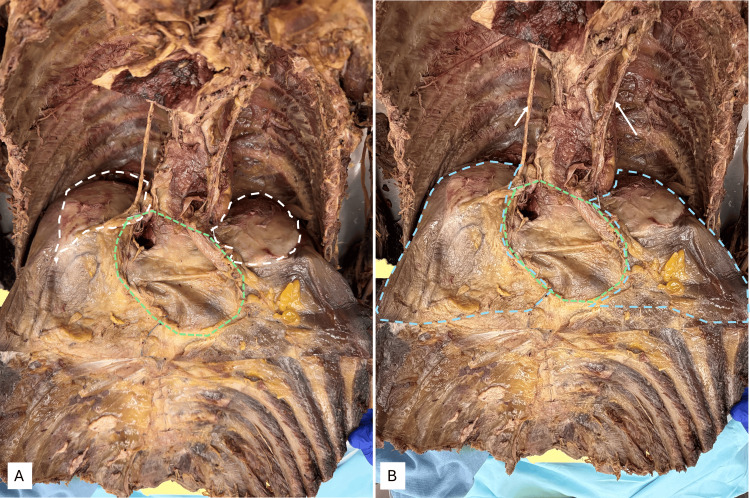
Overview of the diaphragm - image 2 A: Tendinous portion of the diaphragm (green dotted line) and pleural plaques (white dotted lines). B: Tendinous portion of the diaphragm (green dotted line) and muscular portions (blue dotted lines). The arrows show both phrenic nerves

The images below in Figure 3 show a superior view of the thoracic cavity, demonstrating the location of the plaques (white dotted lines), which were indicated by the stars in Figure 2, and the tendinous portion of the diaphragm, indicated by green dotted lines. The image on the right shows the entire muscular portion of the diaphragm outlined by pale blue dotted lines. In these two images, the pleural plaques can be differentiated from the rest of the diaphragmatic parietal pleura by the white and blue dotted lines. The shiny lining of the muscular portion of the diaphragm indicates the normal diaphragmatic pleura.

Measurements

**Figure 4:**
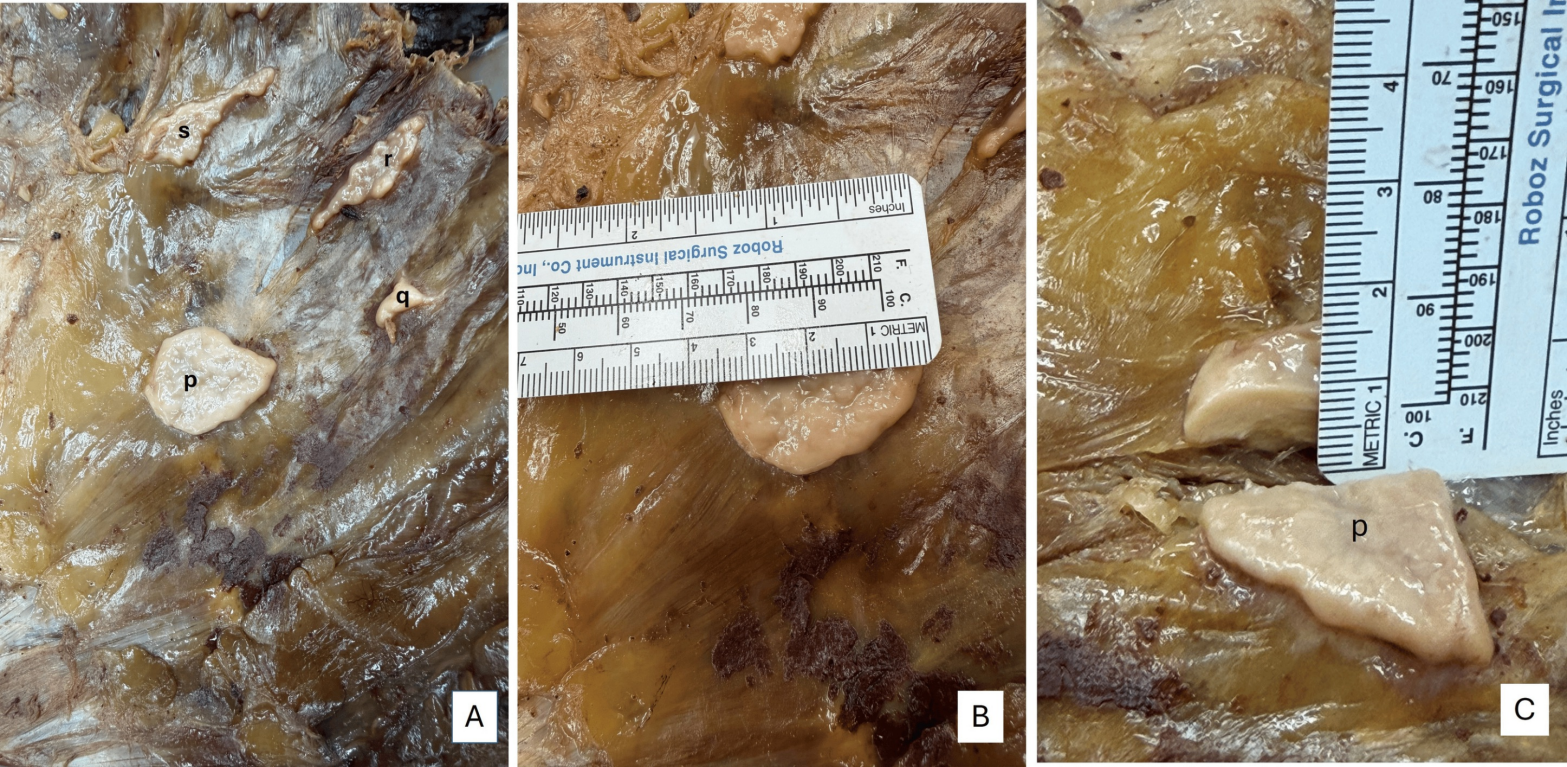
Measurements - image 1 Image A shows plaques (p-s) in the anterior chest wall. Image B shows the horizontal measurement of plaque p. Image C shows the thickness of plaque p

The scattered, isolated plaques on the anterior chest wall were identified and marked as p-s. In Figure 4, the plaque labeled p in image A was the largest and measured 3.5 (horizontal) x 2.5 cm (vertical), with a thickness of about 0.3 cm. A sample was collected for histopathology from plaque labeled p. Samples were not obtained from q, r, and s, as they appeared similar to plaque labeled p, but of smaller diameters.

**Figure 5:**
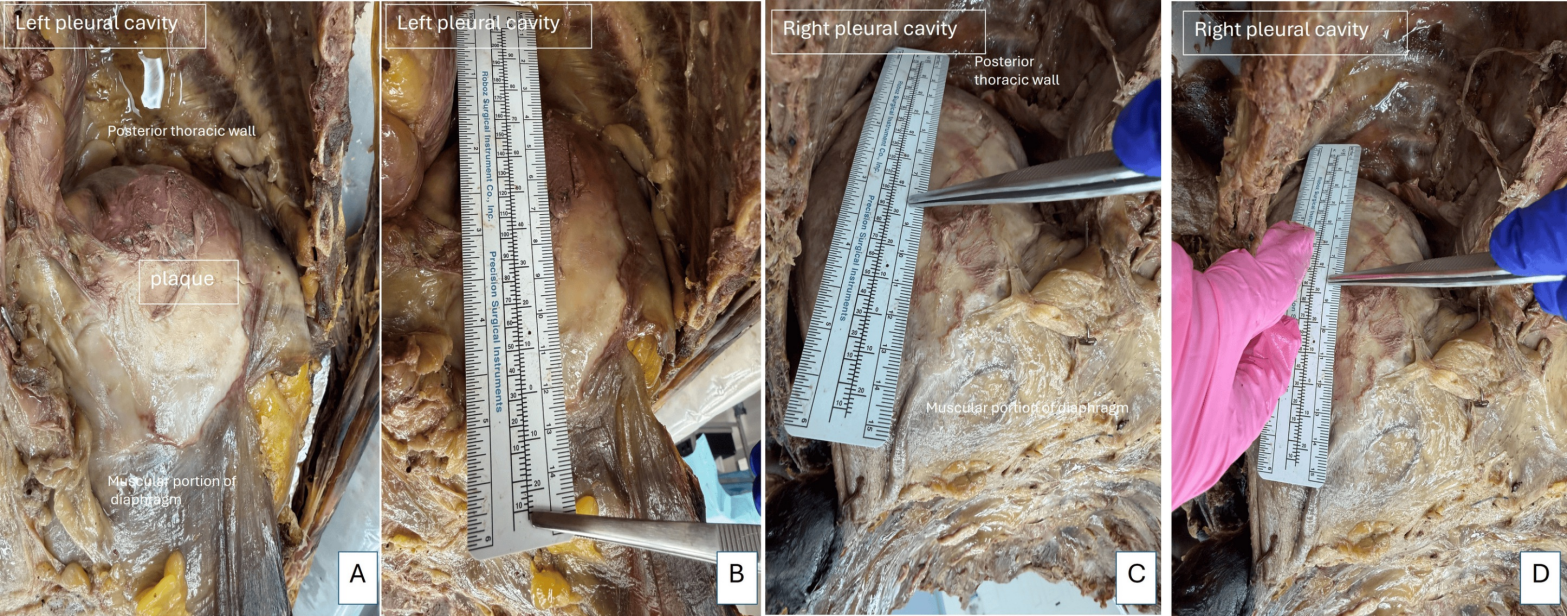
Measurements - image 2 Image A shows the location of the plaque within the left pleural cavity. Image B shows the measurement of the plaque. Images C and D show the locations and measurements of plaque within the right pleural cavity

The larger plaques found on the diaphragmatic aspects were measured (Figure 5) with a surgical ruler. Within the left pleural cavity (Figures 5A, 5B), a whitish plaque was located adjacent to the tendinous portion of the diaphragm, with the following measurements: 12.9 x 6.5 cm; thickness of 0.8 cm. Within the right pleural cavity (Figures 5C, 5D), the whitish plaque was located adjacent to the tendinous portion of the diaphragm, with the following measurements: 13.5 x 10 cm; thickness of 0.8 cm.

**Figure 6:**
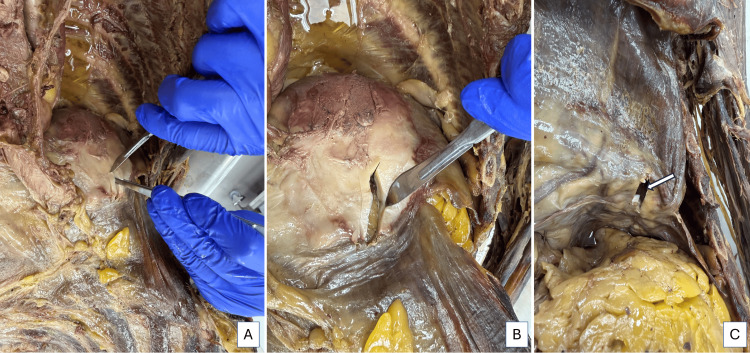
Left pleural cavity and view of the diaphragm from the abdominal cavity Images A and B show the collection of samples from the pleura for analysis. Image C shows the view of the diaphragm from the abdominal cavity

Figures 6A-6B show the approach of the plaque for the acquisition of specimens for histopathological examination (HPE). A small slice of the plaque was removed from the left side, the excision extending through the entire diaphragm. The images show the incision, post-retrieval, and the view of the excised area from the abdominal aspect (Figure 6C). No abnormality was noted in the parietal peritoneum. The pleural plaque could be visualized as a whitish thickening within the extent of the muscular diaphragm. 

**Figure 7:**
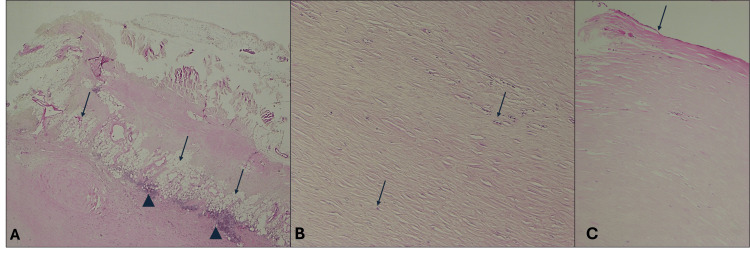
Diaphragmatic and anterior thoracic wall pleural plaques A: Low-power photomicrograph of pleural plaque with adherent adipose tissue, 2X (H&E) stained slide; B: Dense fibrosis within pleural plaque, 10X H&E-stained slide. C: Dense fibrosis with mesothelium lining (arrow), 10X H&E-stained slide

Light micrographs (Figure 7) - obtained from diaphragmatic pleural plaque and stained with hematoxylin and eosin (H & E) - show dense fibrosis with thick collagen extracellular matrix with sparse, hypocellular fibroblasts. Figure 7A shows adherent adipose tissue (arrows) with intervening dense inflammatory infiltrate composed of mononuclear cells (arrowheads). The adipose tissue towards the top was close to the muscular aspect of the diaphragm. Figure 7B shows a higher magnification image of the dense collagen extracellular matrix with hypocellular fibroblasts (arrows). Figure 7C demonstrates a single layer of flat to cuboidal mesothelial cells (arrow) lining the periphery of the pleural plaque towards the pleural cavity. The light micrographs from the specimen obtained from plaque A in Figure 4 revealed similar findings and hence have not been included here.

**Figure 8:**
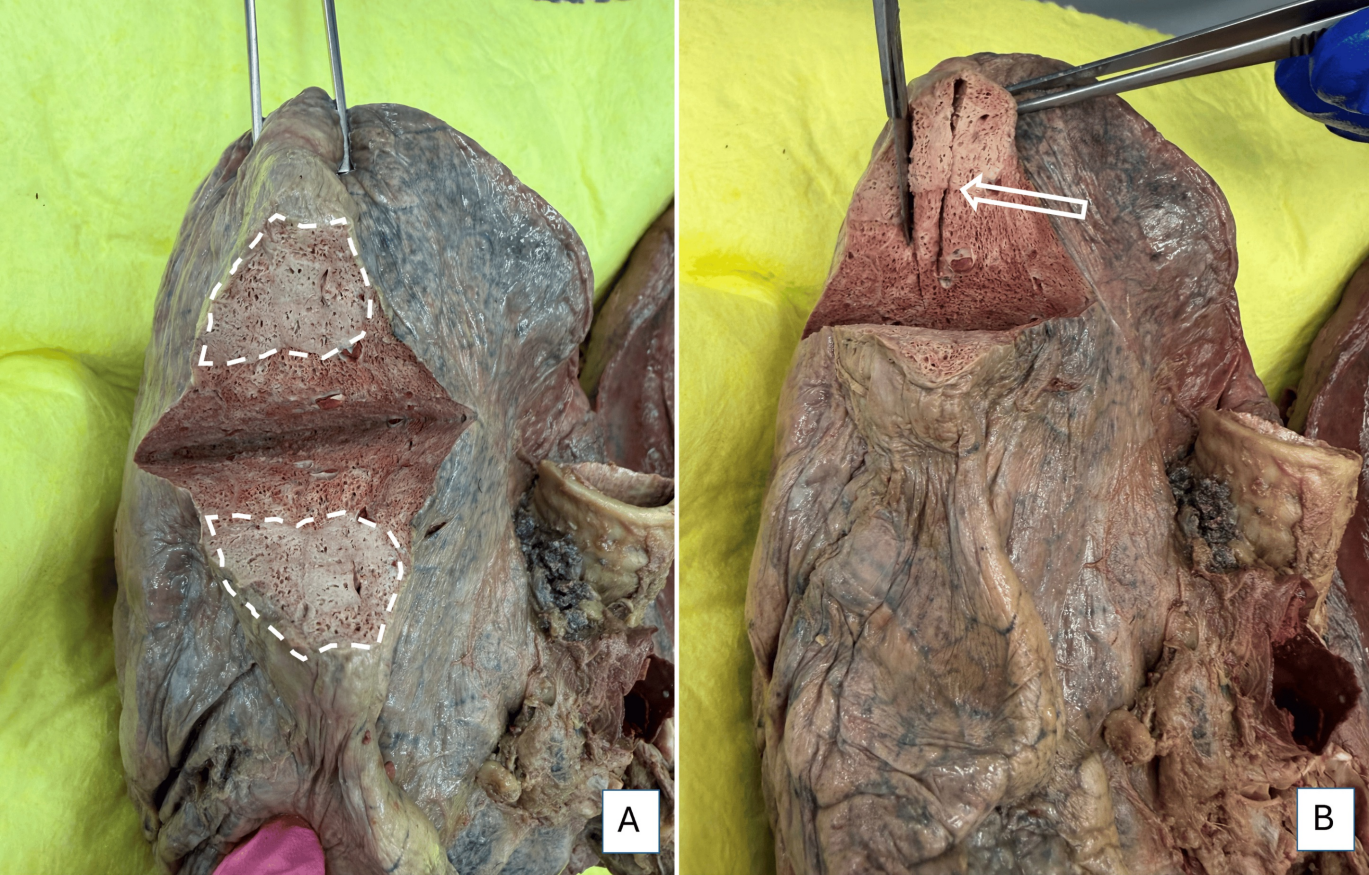
Nodule from the right lung A palpable firm mass of 2.4 x 2.2 x 3.9 cm was noticeable in the right superior lobe. The lung tissue was sliced to reveal the mass (white dotted lines). Area of resection of tissue for HPE is shown in image B, which includes normal alveolar tissue adjacent to the mass (white arrow) HPE: histopathological examination

A nodule from the right lung is presented in Figure 8. A firm palpable nodular area of 2.4 x 2.2 x 3.9 cm was observed in the right superior lobe on the anterior aspect (white dotted line in Figure 8A). The specimen from the nodule was collected through the lung tissue, including areas of normal lung tissue at the periphery (the white arrow). The arrow in Figure 8B points to the junction of the boundaries of the nodule and the normal lung tissue. The specimen included this area to delineate any pathological finding within the nodule from the adjacent normal lung parenchyma.

**Figure 9:**
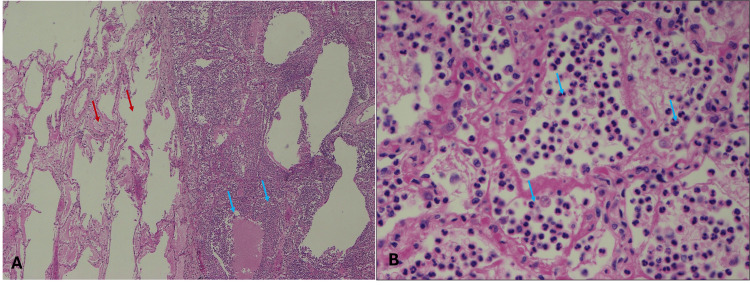
Lung consolidation A: Low-power photomicrograph of the area of lung consolidation (blue arrows) with adjacent normal alveoli within lung parenchyma (red arrows), 4X H&E-stained slide. B: Severe neutrophilic infiltrate within alveolar spaces, 40X H&E-stained slide

Light micrographs (Figure 9) show normal lung parenchyma with healthy alveoli, with an abrupt change to a hypercellular infiltrate within the alveolar spaces. Figure 9A shows unremarkable alveoli and vasculature (red arrows) adjacent to alveolar spaces filled with inflammatory cells, predominantly neutrophils (blue arrows). Figure 9B shows a higher magnification image of the severe neutrophilic infiltrate (some inflammatory cells at blue pointers) within the thin-walled alveoli, suggesting acute pneumonia.

## Discussion

Pleural plaques are the most common manifestation of asbestos-related pleural disease and primarily serve as markers of prior asbestos exposure rather than causes of significant symptoms, developing in roughly 50% of exposed individuals, often decades after exposure. They are usually asymptomatic and incidentally detected on imaging or autopsy, initially appearing as small, well-defined lesions along the parietal pleura, particularly over rib surfaces while sparing the intercostal spaces, and may progressively enlarge, coalesce, and calcify over time [2]. Although asbestos exposure is the classic cause, pleural plaques and calcifications (pleuritis calcarea) may also result from prior infections such as tuberculosis, empyema, trauma, hemothorax, end-stage renal disease, or chronic inflammatory conditions, including systemic lupus erythematosus (SLE) and rheumatoid arthritis, and may occasionally be idiopathic [3].

When extensive, plaques can restrict lung expansion and lead to exertional dyspnea that may progress to dyspnea at rest, with associated dry cough, inspiratory crackles, and, in advanced cases, digital clubbing and respiratory insufficiency. Computed tomography is the most sensitive imaging modality, identifying plaques in about half of patients compared with lower detection rates on chest radiography, while morphologic examination confirms most cases [2]. One study found that substantial cumulative exposure was a strong predictor of minor radiological findings, including pleural plaques or no abnormalities, while 28.5% of patients developed asbestos-related diseases, including mesothelioma [4].

Inflammation of the pleura can lead to replacement of the normal thin membrane with fibrous tissue, which usually resolves once inflammation subsides, though some individuals are left with minor, asymptomatic pleural thickening that does not impair lung function. In rare cases, extensive fibrosis can encase a lung, restrict expansion, impair oxygen uptake, and may require surgical removal of the fibrotic pleura; calcifications may also develop within fibrotic areas [1]. Non-asbestos pleural plaques are often unilateral and may involve the costophrenic angles, whereas asbestos-related plaques are typically bilateral and spare these regions.

Pulmonary asbestosis generally remains asymptomatic for 20-30 years after exposure, depending on exposure intensity, while pleural disease often appears earlier, with benign asbestos-related pleural effusions occurring within 15 years. When symptoms develop, they usually begin with exertional dyspnea progressing to dyspnea at rest, accompanied by dry cough, end-inspiratory crackles, and sometimes severe thoracic pain related to pleural involvement; wheezing and sputum production are uncommon unless attributable to smoking. Advanced disease may feature digital clubbing, respiratory insufficiency, pulmonary hypertension, and an increased risk of lung cancer among asbestos-exposed workers [2].

Although pleural plaques are mostly associated with prior asbestos exposure, the literature identifies numerous alternative environmental and medical contributors. Non-asbestos mineral fibers, including erionite, wollastonite, mica, kaolin, and some man-made vitreous fibers, have been linked to plaque formation in occupational and environmental settings, while medical conditions such as tuberculosis, empyema, hemothorax, inflammatory pleurisy with effusion, chest trauma, and rib fractures can produce similar pleural changes. Systemic and autoimmune diseases, including rheumatoid arthritis, scleroderma, and SLE, as well as chronic renal failure, talc pleurodesis, and malignancies such as myeloma, have also been associated with post-inflammatory pleural fibrosis [3].

Evidence for many of these associations derives largely from case reports and small studies, limiting causal certainty and underscoring the importance of obtaining a comprehensive occupational, environmental, and medical history when determining etiology [5]. Illustratively, a reported case of a 73-year-old man with bilateral calcified pleural plaques and pleural effusion demonstrated fibrotic and hyalinized plaques without detectable tuberculosis on biopsy, yet clinical response to antituberculosis therapy ultimately established tuberculosis as the underlying cause [6]. Additional reports describe autoimmune-mediated pleural fibrosis around silica-laden sites, including lupus pleuritis associated with silica exposure and rheumatoid arthritis, in which pleural plaques and thickening may occur commonly and often remain asymptomatic [7,8].

Gross appearance and histopathology

Autopsy and histological studies of pleural plaques consistently demonstrate well-circumscribed lesions composed of dense, hyalinized, acellular collagen, often arranged in a characteristic basket weave pattern and frequently showing calcification [2,9]. Gross examination may reveal parietal pleural plaques, while histology typically shows dense collagen without significant vascularity, covered on the pleural surface by a single layer of mesothelial cells [10]. Lung tissue in affected cases may demonstrate associated interstitial fibrosis and asbestos bodies; however, asbestos fibers are notably absent within the plaques themselves, even on electron microscopy. Light microscopic hematoxylin and eosin-stained sections at varying magnifications highlight these dense collagenous plaques and are often used diagnostically to distinguish pleural plaques from other entities such as fibrosing pleuritis or desmoplastic mesothelioma [9,11].

Images of fibrous pleural plaques measuring 3-6 cm in diameter with smooth margins and a hard surface were found to be spread out on the left parietal pleura and chest surface of the thoracic diaphragm. The histological analysis revealed fibrotic, hyalinized structures with focal calcifications [12]. The histological findings from our cadaveric study show a large amount of dense, irregularly arranged type I collagen fibers with fibroblasts and a few scattered inflammatory cells, with minimal ground substance in the extracellular matrix. The lung parenchyma showed no evidence of asbestos crystals. Inflammatory cells were also present within a firm nodular area of lung parenchyma that was palpable in the anterior border of the right upper lobe. The collagen within the plaque exhibited a basket-weave pattern consistent with that described in pleural plaques [11].

Gross images taken at autopsies describe the pleural plaques as discrete, raised lesions distributed bilaterally in approximately 85% of cases, most commonly along the posterolateral parietal pleura adjacent to the fifth through eighth ribs. With time, these asbestos-related plaques may accumulate calcium and acquire a characteristic white to yellow "candle wax dripping" appearance on gross examination. Histologically, they are described as paucicellular lesions composed of dense, hyalinized collagen arranged in a basket weave pattern [11,13]. Gross documentation of pleural plaques remains relatively uncommon in the literature, but reported images demonstrate either isolated plaques or patchy involvement of the costal pleural surfaces, including a classic case of a shipyard worker with parietal pleural plaques and associated pulmonary interstitial fibrosis containing asbestos bodies identified at postmortem examination [10].

Radiological appearance of pleural plaques

Imaging characterizes pleural plaques as benign, fibrous lesions composed of collagen deposits on the parietal pleura, most often reflecting prior asbestos exposure. They frequently demonstrate calcification and appear on chest radiography or computed tomography as dense, irregularly marginated opacities with a characteristic bilateral, scalloped configuration, commonly referred to as the “holly leaf” sign. These high‑attenuation plaques are typically distributed along the diaphragm and lateral chest wall, preferentially involving the parietal pleura while sparing the costophrenic angles. Their distinctive morphology and distribution on imaging are key features that aid in recognizing asbestos‑related pleural disease [14].

**Figure 10:**
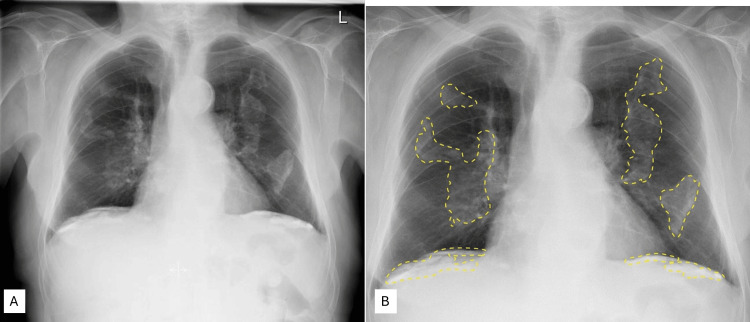
Chest radiographs of a 65-year-old male Image A shows calcified plaques on the anterior chest wall and the diaphragmatic surface. Image B shows the same chest X-ray, where the plaques are highlighted with yellow dotted lines User-generated image content in accordance with the CC License; https://radiopaedia.org/cases/12388 (rID number 12388)

Figure 10 shows chest radiographs of a 65-year-old male who had symptoms of breathlessness and increased sputum production. There are extensive calcified plaques, reported to be in the parietal pleura on the anterior chest wall and diaphragm. Plain chest X-ray (CXR) with extensive bilateral pleural plaques is shown in Figure 10A, and the same plaques have been highlighted with yellow dotted lines in Figure 10B [14].

Cadaveric findings

Based on the discussion of the donor’s occupation and the known etiology and appearance of such plaques, they most likely resemble those that develop due to asbestos exposure over the years. However, this extensive distribution and thickness of the plaques suggest a long-standing process over many years, and as a result, he may have remained asymptomatic for a prolonged period [2]. The plaques would have appeared on chest radiograph as depicted in Figure 10 in the diaphragmatic areas bilaterally and the anterior chest wall [14]. Histopathology has shown similar features of collagen accumulation and calcification as described in other studies of plaques with a basket weave appearance [11,13]. However, the gross images from the cadaver in our study are more extensive than those reported in the literature, where plaques are often described as smaller lesions with a “candle wax drippings” appearance [11,13]. In addition, there has been limited documentation of precise measurements of such plaques in prior reports.

Limitations of the study

As the study involved a cadaver with a cause of death being a glioblastoma of approximately one month's duration, there is no definitive way to establish the etiology of the pleural plaques beyond his occupation as a steam fitter. There were no reported underlying cardiovascular or pulmonary conditions. Although histopathology confirmed a calcified pleural plaque associated with mild inflammation in adjacent areas and the presence of acute pneumonia within lung tissue, further information regarding prior infections or treatment for conditions such as tuberculosis, rheumatoid arthritis, SLE, or malignant disease is not available in this case.

## Conclusions

Bilateral pleural thickening has been an uncommon finding during cadaveric dissections in the preclinical phase of the medical curriculum at GCSOM. Encountering such pathology naturally prompts curiosity regarding the underlying disease processes that may have contributed to its development, the morbidity associated with these conditions, and the treatments that might have been employed to alleviate symptoms during life, if present. In cases with no corroborating evidence of severe interstitial lung disease secondary to asbestosis or findings suggestive of mesothelioma, determining the etiology of extensive bilateral pleural plaque formation becomes particularly challenging. Given the limited availability of gross anatomical images, along with scarce documentation of measurements and descriptions of the texture of such extensive bilateral plaques and corresponding histological illustrations in the published literature, we aim to disseminate these observations more broadly to contribute to and enrich the current educational resources, support future research, and deepen understanding of pleural pathology observed in anatomical studies.
